# JNS Review Recognition 2020

**DOI:** 10.1017/jns.2020.48

**Published:** 2020-11-23

The Editorial Board of the *Journal of Nutritional Science* would like to thank the following for their contribution as peer reviewers in 2020:

Edyta Adamska
Folasade Adebayo
Demetrius Albanes
Meshari Alkoot
Naser Alsharairi
Giannis Arnaoutis
Zahra Bahadoran
Emma Baldry
Guillermina Barril-Cuadrado
Eirini Bathrellou
Carolina Berchieri- Ronchi
Anne Bjerregaard
Petra Brembeck
Magritt Brustad
Jennifer Carter
Muhammad Chacher
Lieselotte Cloetens
Peter Curtis
Sara da Silva
Luana Azevedo de Aquino
Bethelhem Debela
Hans Demmelmair
Arvind Diwan
Saeid Doaei
Gudina Egata
Arja Erkkilä
Edna Gamboa
Michael Georgoulis
Admasu Gizaw
Beatriz Gonçalves
Bartira Gorgulho
Horst Göring
Jessica Gwin
Stephen Hennigar
Javad Heshmati
Andrew Hill
Iva Hojsak
Paul Hulshof
Aiko Hyakutake
Francois Iris
Patricia Jaime
Niina Kaartinen
Russell Kabir
Satish Kalhan
Nitin Kapoor
J. Philip Karl
Maryam Khan
James King
Mirit Kisner
David Kitts
Yoko Komada
Patrícia Lisboa
Tahereh Mahammadabadi
Simonette Mallard
Eirini Mamalaki
Y. Fabiola Marquez-Sandoval
Koutatsu Maruyama
Velimir Matkovic
Walter Mihatsch
Arlene Mitchell
Maedeh Moradi
Karen Murphy
Tzortzis Nomikos
Meghan O'Neill
Archana Panche
Jaqueline Pereira
Marcos Pereira-Santos
Elsa Pinto
Alex Rafacho
Alanderson Ramalho
Stina Ramne
Elisabet Rothenberg
Lisa Ryan
Aki Saito
Jonathan Scott
Harbindar Singh
Chelsea Singleton
Regina Spadari-Bratfisch
Zhihong Sun
Giovanni Tarantino
Lílian Gonçalves Teixeira
Paul Trayhurn
Konstantinos Tziomalos
Pedro Urriola
Simone Vargas
Clemens von Schacky
Yeli Wang
Leigh Ward
Kurt Widhalm
Lynda Williams
Katsuhiko Yokoi
Katherine Younger
Jing Yuan

